# De novo synthesize of bile acids in pulmonary arterial hypertension lung

**DOI:** 10.1007/s11306-014-0653-y

**Published:** 2014-04-11

**Authors:** Yidan D. Zhao, Hana Z. H. Yun, Jenny Peng, Li Yin, Lei Chu, Licun Wu, Ryan Michalek, Mingyao Liu, Shaf Keshavjee, Thomas Waddell, John Granton, Marc de Perrot

**Affiliations:** 1 Latner Thoracic Surgery Research Laboratories, Division of Thoracic Surgery, University of Toronto, Toronto, ON Canada; 2 Metabolon, Incorporated, 617 Davis Drive, Durham, NC 27713 USA; 3 Clinical Studies Resource Centre, Toronto General Hospital, University Health Network, University of Toronto, Toronto, ON Canada; 4 MaRS Centre, Toronto Medical Discovery Tower, 2nd Floor Rm 2-817, 101 College Street, Toronto, ON M5G 1L7 Canada

**Keywords:** Bile acid pathway, Pulmonary arterial hypertension, Lung

## Abstract

Although multiple, complex molecular studies have been done for understanding the development and progression of pulmonary hypertension (PAH), little is known about the metabolic heterogeneity of PAH. Using a combination of high-throughput liquid-and-gas-chromatography-based mass spectrometry, we found bile acid metabolites, which are normally product derivatives of the liver and gallbladder, were highly increased in the PAH lung. Microarray showed that the gene encoding cytochrome P450 7B1 (CYP7B1), an isozyme for bile acid synthesis, was highly expressed in the PAH lung compared with the control. CYP7B1 protein was found to be primarily localized on pulmonary vascular endothelial cells suggesting de novo bile acid synthesis may be involved in the development of PAH. Here, by profiling the metabolomic heterogeneity of the PAH lung, we reveal a newly discovered pathogenesis mechanism of PAH.

**Electronic supplementary material:**

The online version of this article (doi:10.1007/s11306-014-0653-y) contains supplementary material, which is available to authorized users.

## Introduction

Pulmonary arterial hypertension (PAH) is a severe vascular disease characterized by persistent precapillary pulmonary hypertension (PH) (Stacher et al. 2012; International PPHC et al. 2000; Zhao et al. 2002; Fujiwara et al. 2008; Nasim et al. 2011; Olschewski 2010; Bogaard et al. 2012; MMea and 2013), which can be either be idiopathic (sporadic-90 %, familial-10 %). PAH can also be a complication associated with other conditions such as connective tissue disease, congenital heart disease, anorexigen use (dexfenfluramine), portal hypertension, and human immunodeficiency virus (Stacher et al. 2012; International PPHC, Lane KB, Machado RD, Pauciulo MW, Thomson JR, et al. 2000; MMea et al. 2013). Evidence in the literature suggests that metabolic pathway abnormalities characterize and may play a significant role in the development and progression of PAH (Fessel et al. 2012). For example, pulmonary arterial endothelial cells (PAECs) in PAH share similar hyperproliferative characteristics as malignant tumor transformation that is accompanied by significant metabolic shifts to support anabolic growth and energy metabolism (Xu et al. 2005; Chen et al. 2007). Moreover, it has been shown that mitochondrial oxidative phosphorylation with glucose uptake and utilization occurs in PAEC development. Significant elevation of hemoglobin has been found in the PAH sample group without a history of diabetes or any other obvious metabolic diseases, indicating the impairment of whole-body glucose homeostasis in PAH (Pugh et al. 2011; Hansmann et al. 2007; Archer et al. 2010). Additionally, vascular changes under chronic hypoxic condition has been directly linked to an imbalance between glycolysis, glucose oxidation, and fatty acid oxidation (Sutendra et al. 2010), while in vitro pulmonary arterial endothelial cell culture with disruption of the Bone Morphogenetic Protein Receptor II (BMPRII) gene showed significant metabolomic changes (Fessel et al. 2012). Our recent work showed that disrupted glycolysis, increased TCA cycle, and fatty acid metabolites with altered oxidation pathways exited in the human PAH lung, indicating that PAH has specific metabolic pathways contributing to abnormal ATP synthesis for the vascular remodeling process in pulmonary hypertension (Zhao et al. 2014). Collectively, in vitro, human and animal models suggest that multiple metabolic pathways are reprogrammed during PAH vascular remolding and that metabolic heterogeneity may play an important role in both ATP energy supply and the molecular pathogenesis of pulmonary hypertension. Here, we provide direct evidence of a novel increase in bile acid metabolites in PAH lung tissue associated with the elevated expression of bile acid synthesis related transcripts, indicating de novo synthesis of bile acids may characterize and contribute to the pathogenesis of PAH.

## Materials and methods

Global biochemical profiles were determined in human lung tissue and compared across 8 normal (47 ± 15 years of age, 4 females) and 8 pulmonary arterial hypertension patients (40 ± 12 years of age, 5 females). Eligibility criteria included end stage PAH patients who went through lung transplantation. Lung samples were obtained from the recipient lung at the time of lung transplantation. Control lung samples were obtained from normal tissue of cancer patients undergoing surgery (lobectomy). Biospecimens and associated clinical data related to the study were collected with written consent from the University Health Network and approved by the Internal Review Board. Unbiased metabolomic profiling using liquid/gas chromatography coupled to mass spectrometry (LC/GC–MS) was performed as described (Reitman et al. 2011; Evans et al. 2009). The detail procedure of metabolic analysis has been documented in the *Supplement data*.

### Transcriptomic analysis

mRNA samples from the normal (n = 8) and native PAH lungs (n = 8) were isolated as described (Zhao et al. 2014). Bile acid related profiles were compared between a control group and samples with idiopathic pulmonary arterial hypertension. Briefly, the total RNA analysis in lung tissues was performed using Trizol extraction according to the manufacturer’s instructions. Biotinylated cRNA was prepared according to the standard Affymetrix protocol (Expression Analysis). Following fragmentation, cRNA were hybridized on GeneChip Genome Array. GeneChips were scanned using the HuGene-1_0-st-v1 GeneArray Scanner G2500A. The data were analyzed with Partek Genomics Suite 6.6 using the Affymetrix default analysis settings and global scaling as the normalization method. The value definition was set up using Partek Genomics Suite 6.6. Significantly changed genes were determined by *t* test with a false discovery rate of two fold. The data base has been submitted to NCBI/GEO and has been approved and assigned a GEO accession number GSE53408.

### Immunoblotting

Protein concentrations were determined using the BCA protein assay (Pierce, IL, USA). Equal amounts of the protein lysates were separated by SDS-PAGE and transferred onto nitrocellulose membranes. The membranes were incubated for overnight at 4 °C with the following antibodies from Abcam^R^: anti-CYP7B1(1:1,000). After wash with TBS-Tween, the blots were incubated for 60 min at room temperature with horseradish peroxidase-conjugated antibodies, respectively: anti-rabbit antibody (1:15,000; Sigma-Aldrich, St. Louis, MO). Signals from immunoreactive bands were visualized by fluorography using an ECL reagent (Pierce). The intensity of individual bands in immunoblots were quantified using the NIH Image program.

### Immunohistochemistry

The sections of both PAH and normal lung tissue were fixed for 4 h at room temperature with PBS made of 4 % formaldehyde, permeabilized for 30 min in Triton X-100 (0.5 % in PBS), and incubated with 5 % nonfat skim milk in PBS for 90 min. Sections were incubated for 180 min at room temperature with antibodies for anti-CYP7B1 (1:1,000). The sections were then incubated with biotinylated secondary antibody and visualized with DAB. Stained cells and sections were visualized with the Zeiss LSM 510 confocal microscope.

## Results and discussion

**Fig. 1:**
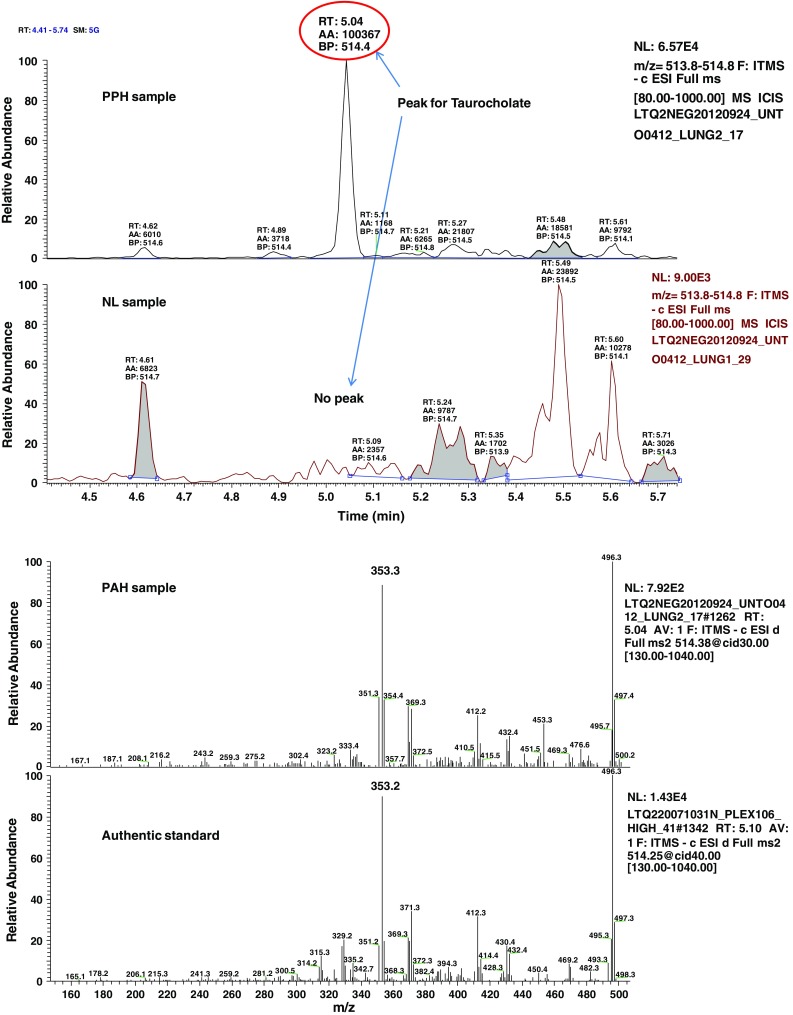
MS/MS fragmentation spectrum of taurocholate in control and PAH lung. *Top panel* shows a representative negative ion, selected ion chromatogram (SIC) for taurocholate (*m/z* 514.3) in normal (NL) and pulmonary hypertension (PAH) lung tissue. Taurocholate compound identification relied on confirmed experimental MS/MS fragmentation spectrum matched to the authenticated taurocholate standard, run separately (*bottom panel*). Limited peak detection was observed in NL samples

**Fig. 2:**
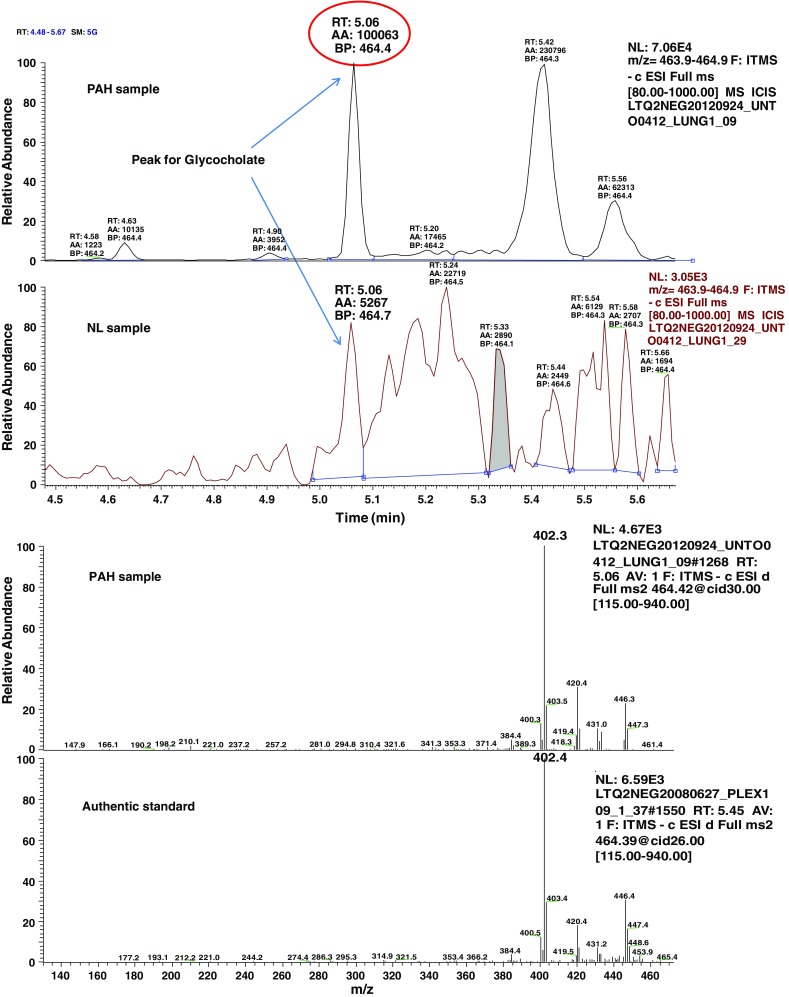
MS/MS fragmentation spectrum of glycocholate in control and PAH lung. Representative negative ion is selected ion chromatogram (SIC) for glycocholate (*m/z* 464.4) in normal (NL) and pulmonary hypertension (PAH) lung tissue (*top panel*). Glycocholate compound identification relied on confirmed experimental MS/MS fragmentation spectrum matched to the authenticated glycocholate standard, run separately (*bottom panel*)

**Fig. 3:**
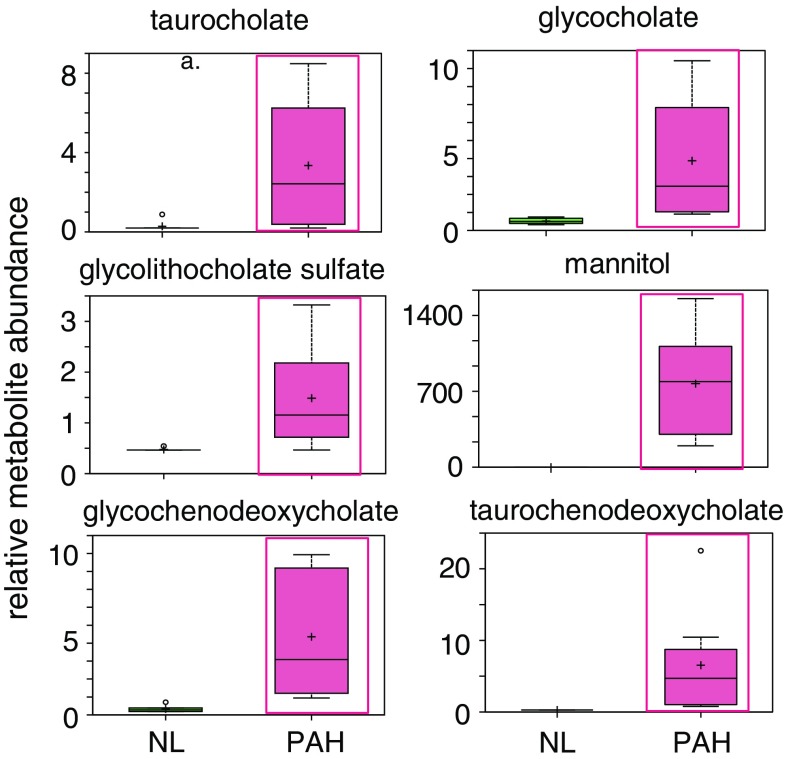
PAH lung has a unique bile acids metabolic pathway. Intermediates in the bile acids pathway revealed significantly elevated levels of multiple glycine and taurine conjugated bile acids in the PAH lung. Data for normal lung (NL, n = 8) are represented in *green boxes*, while data for pulmonary hypertension lung (n = 8) are shown in *pink boxes*. Quantities are in relative arbitrary units specific to the internal standards for each quantified metabolite and normalized to protein concentration (PAH with *red frame* indicates *p < 0.05 compared to NL

**Fig. 4:**
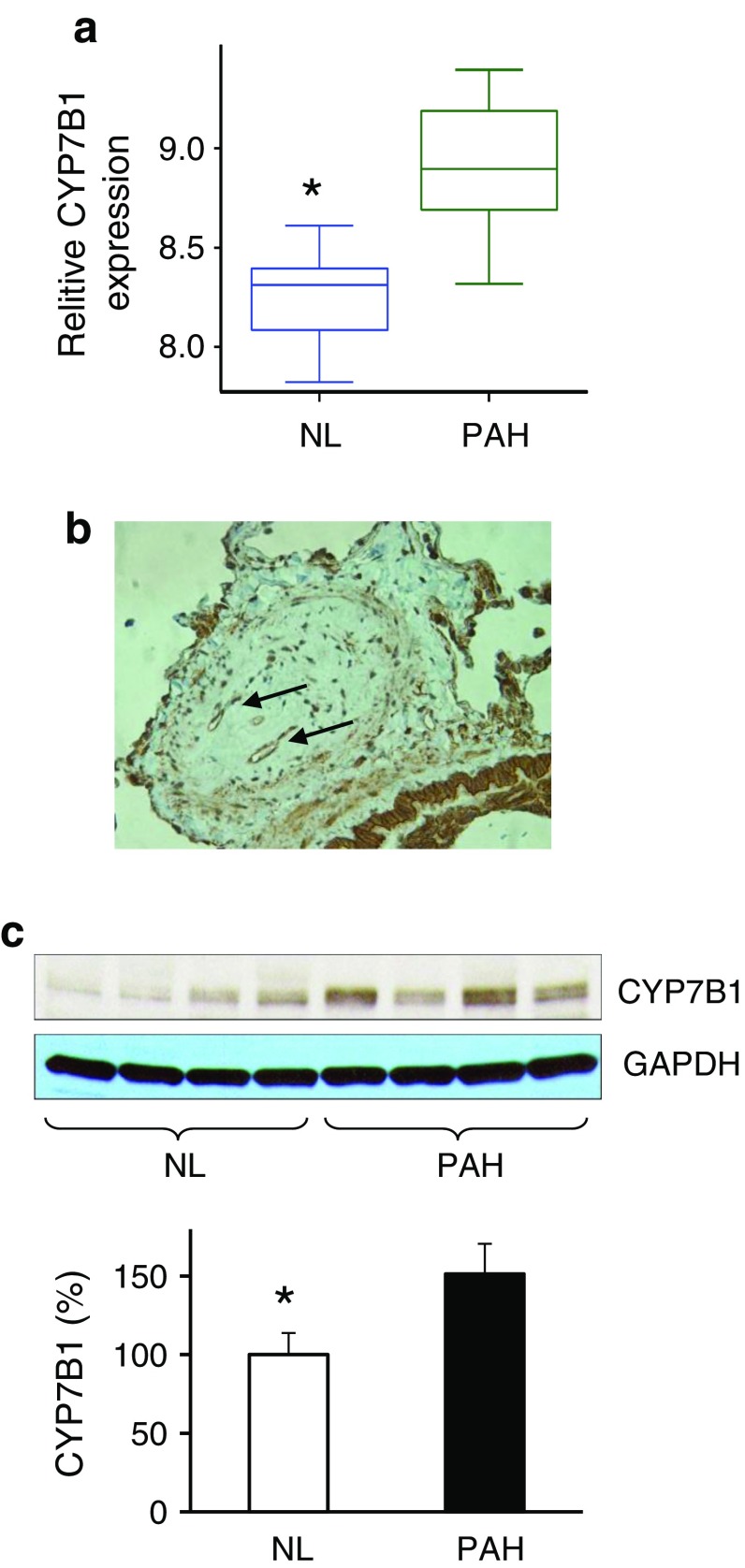
**a** Microarray data showed that the gene encoding cytochrome P450, family 7, subfamily B, polypeptide 1 (Oxysterol 7α-hydroxylase) was significantly highly expressed in PAH lung. (*p* = 0.000187299). **b** Western blot analysis of CYP7B1 expression in normal and PAH lungs. Lung lysate was loaded and immunoblotted with antibody against CYP7B1 and GAPDH (loading control). Consistent with a significant increase of CYP7B1 gene expression in PAH, the enzyme protein for CYP7B1 (37KD) was significantly increased in PAH lungs compared with NL lungs. Densitometric analysis of CYP7B1 was normalized to the intensity of the respective GAPDH band. Data are expressed as mean ± SD (n = 4). *p < 0.05 versus NL. **c** CYP7B1 positive immunostaining in newly formed small blood vessels (*arrows*) in the plexiform lesions of occluded pulmonary small vessel in PAH lung. Representative micrographs of immunostaining of PAH lung sections are shown with anti–CYP7B1 in the pulmonary vascular endothelial cells. (ratio 1:200)

**Fig. 5:**
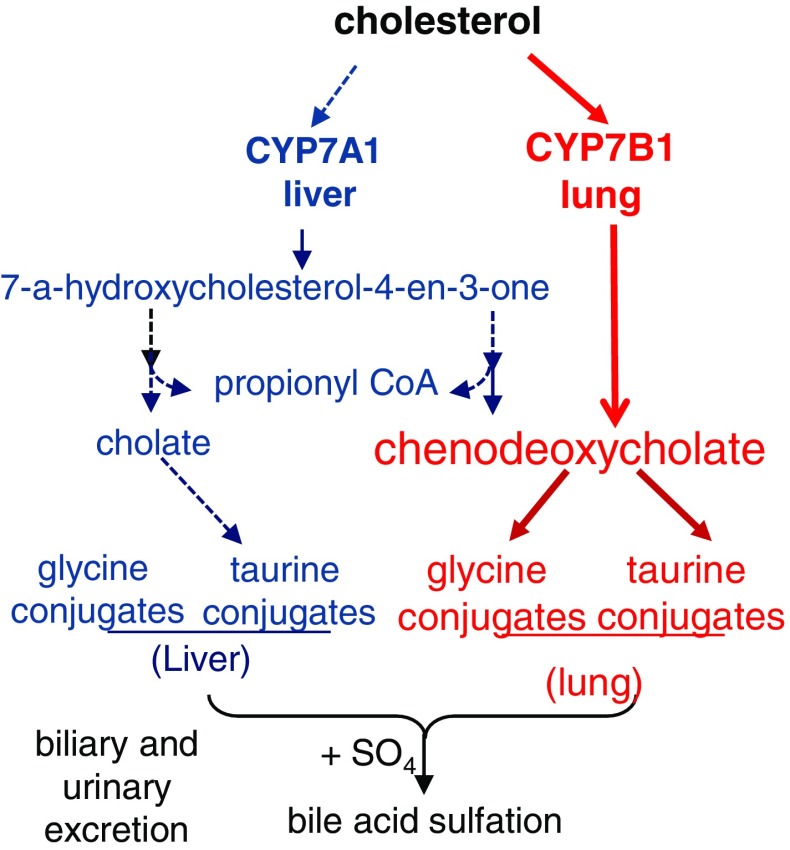
Major intermediates in the classical bile acids pathway through Cholesterol 7 alpha-hydroxylase, also known or cytochrome P450 7A1 (CYP7A1), are shown in *blue*. Our finding suggests that PAH lung has a specific bile acids pathway though CYP7B1, as shown in *red*

We explored and characterized the metabolomic signature of pulmonary hypertension (PAH) to enhance our understanding of disease progression. Using untargeted metabolic profiling, we found that PAH lung (n = 8) possessed significantly higher levels of multiple bile acid metabolites, including the primary bile acids taurocholate (Fig. 1), glycocholate (Fig. 2), taurochenodeoxycholate, and glycochenodeoxycholate (Fig. 3). Bile acids are normally synthesized in the liver and gallbladder from cholesterol by 7-alpha-hydroxylase, also called cytochrome P450 (CYP7A1), as a rate-limiting enzyme in the synthesis of bile acid via the classic pathway (Nishimoto et al. 1993; Cohen et al. 1992; Crestani et al. 1993; Wang and Chiang 1994). Although the presence of bile acids in lung tissue may partially reflect reflux in these patient (D’Ovidio et al. 2005; Blondeau et al. 2009), microarray analysis surprisingly revealed that the gene encoding cytochrome P450 B1 (CYP7B1), but not CYP7A1, had a significantly higher expression in PAH lung (Fig. 4a). This finding was also confirmed by Real time RTPCR. Further molecular analysis using Western blot showed that the expression of CYP7B1enzyme was higher in PAH lung (Fig. 4b). These results suggest that increased bile acid metabolites may not solely be due to reflux from the esophagus (D’Ovidio et al. 2005; Blondeau et al. 2009) but come from the lung itself. Thus, PAH lung tissue may have the capacity for de novo synthesis of bile acids. Notably, increased bile acids metabolites could potentially serve as biomarkers for disease progression. By applying immunohistochemistry, CYP7B1positive immunostaining was found in pulmonary vascular endothelial cells, specifically in newly formed vascular endothelial cells in plexiform lesions of occluded pulmonary arteries (Fig. 4c), suggesting that CYP7B1may also be involved in the vasculogenesis during the vascular remodeling process of PAH (Fig. 5). This hypothesis needs to be further tested by additional functional analyses. In summary, we have shown direct evidence that a de novo synthesis of bile acid may be involved in pathogenesis of PAH, suggesting that bile acids in lavage fluid may serve as ideal biomarkers for the diagnosis and prognosis of PAH.

## Electronic supplementary material

Below is the link to the electronic supplementary material.

Supplementary material 1 (DOCX 14 kb)
